# Predictive Risk-scoring Model For Central Lymph Node Metastasis and Predictors of Recurrence in Papillary Thyroid Carcinoma

**DOI:** 10.1038/s41598-019-55991-1

**Published:** 2020-01-20

**Authors:** Lie-hao Jiang, Ke-xin Yin, Qing-liang Wen, Chao Chen, Ming-hua Ge, Zhuo Tan

**Affiliations:** 1 Department of Head, Neck and Thyroid Surgery, Zhejiang Provincial People’s Hospital, People’s Hospital of Hangzhou Medical College, 158 Shangtang Road, Xiacheng District, Hangzhou, 310014 China; 2 Department of Head and Neck Surgery, Zhejiang Cancer Hospital, 1 East Banshan Road, Gongshu District, Hangzhou, 310016 China; 3 Second Clinical Medical College, Zhejiang Chinese Medical University, 548 Binwen Road, Binjiang District, Hangzhou, 310022 China

**Keywords:** Risk factors, Surgical oncology, Head and neck cancer

## Abstract

There are about half of papillary thyroid carcinoma (PTC) patients with the experience of central lymph node metastasis (CLNM), while the model to predict high-risk groups of CLNM from PTC patients is uncertain. The aim of this study was to evaluate candidate risk factors of CLNM and identify risk factors of recurrence to guide the postoperative therapeutic decision and follow-up for physicians and patients.A total of 4107 patients(4884 lesions) who underwent lymph node dissection in two hospitals from 2005 to 2014 were evaluated. CLNM risk was stratified and a risk-scoring model was developed on the basis of the identified independent risk factors for CLNM. Cox’s proportional hazards regression model was used to investigate the risk factors for recurrence.CLNM was proved in 37.96% (1559/4107) of patients and 33.96% (1659/4884) of lesions. In the multivariate analysis, Male, Age ≤35 years, Tumor size >0.5 cm,Lobe dissemination (+), Psammoma body (+), Multifocality and Capsule invasion (+) were independent risk predictors of CLNM (P  < 0.01). A 14-point risk-scoring model was built to predict the stratified CLNM in PTC patients and the area under receiver operating characteristic curve of the model for the prediction of CLNM was 0.672 (95% CI: 0.656–0.688) (P < 0.01). COX regression model showed that Tumor size >0.5 cm, Lobe dissemination (+), Multifocality and CLNM were significant risk factors associated with poor outcomes. The research suggested that prophylactic CLN dissection could be performed in patients with total score ≥4 according to the risk-scoring model, and more aggressive treatment and more frequent follow-up should be considered for patients with Tumor size >0.5 cm, Lobe dissemination (+), Multifocality and CLNM.

## Introduction

In current years, the global prevalence of thyroid cancer has increased swiftly. In 2012, thyroid cancer even has exceeded breast carcinoma as the most prevalent carcinoma of women in Hangzhou, China^1^. Papillary thyroid carcinoma (PTC) is the most well-known type of thyroid cancer, making up about 85% of thyroid cancer, and constituting 1% of whole human malignant tumors^2,3^. Notwithstanding the extraordinary rate of the 5-year disease-specific survival rate in the excellent prognosis of PTC, which usually can reach 99%, half of PTC patients have suffered from cervical lymph node metastasis^4^. Even in the clinical lymph node negative patients, the rate of cervical lymph node metastasis ranges from 20–50% in different studies^5,6^.

There is still no uniform professional guidelines for cervical lymph node dissection and whether prophylactic cervical lymph node dissection is needed remains controversial. Central compartment was regarded as the most common compartment involved and the American Thyroid Association (ATA) Guidelines recommend that prophylactic central lymph node dissection can be taken into consideration, particularly for the patients who have advanced primary tumors (T3 or T4), and at the meantime ATA does not promote the prophylactic lateral lymph node dissection^7^. Some scholars considered that a prophylactic central lymph node (CLN) dissection alone for thyroid carcinoma may increase the complications and has weak connection with improve survival^8^. However, advanced proof from a large scale nested case-control study proposed that patients with lymph node metastasis experienced a higher mortality, and the incomplete surgical excision was a primary cause for the heightened mortality in PTC patients^3^.

Currently, due to the limitation of clinical examinations, it is hard to find subclinical lymph node metastasis in PTC patients, which may cause incomplete clinical treatment and seriously threaten the health of the patients. Therefore, it has become increasingly important for us to discover appropriate clinical and pathological predictors of lymph node metastasis to guide treatment decisions. Hitherto, a number of possible risk factors have been reported, while the results differ from each other and further investigation is warranted^9,10^.

This study aimed to retrospectively analyze the clinical and pathological data of PTC sufferers diagnosed between 2005 and 2014 to estimate the applicant risk factors of CLNM and create a model based on those factors to predict high-risk groups of CLNM from PTC patients. We also gathered data on prognosis in order to recognize risk factors of recurrence, which may offer guidance on the postoperative therapeutic decision and follow-up for physicians and patients.

## Materials and Methods

### Patient selection

This retrospective study was approved by the Ethics Committee of Zhejiang Cancer Hospital and the Ethics Committee of Zhejiang Provincial People's Hospital.The informed consent was obtained from the patients.The study was performed in accordance with established national and institutional ethical guidelines on the human participants and the use of tissue samples for research. A total of 4107 patients(4884 lesions)who were first treated in the two hospitals between January 2005 and December 2014 were evaluated retrospectively. All the patients were diagnosed as PTC on pathology. Patients with one or more of the following will be excluded: i) patients with previous thyroid resection at another institution; ii) patients with other malignancies; iii) patients with a history of neck surgery for other diseases;

### Preoperative ultrasonography examination and Surgical strategy

All patients in the study underwent ultrasonography (US) examination pre-operation to determine the lymph node status. Tumor number was as well as based on preoperative US examination and lesions were divided into Solitary group with only one nodule or Multifocality group with more than one nodule.

Total thyroidectomy and bilateral CLN dissection was performed in the patients with bilateral PTC, while the patients with unilateral PTC underwent total thyroidectomy or unilateral lobectomy plus isthmusectomy and ipsilateral CLN dissection depending on the condition of the primary tumor. Total thyroidectomy plus isthmusectomy might be considered when unilateral PTC patients met one or more of following conditions: (i) tumor size >4 cm; (ii) multifocal in one lobe; (iii) extrathyroid invasion or (iv) distant metastasis, according to the guidelines of Chinese Thyroid Association. All the patients underwent ipsilateral prophylactic CLN dissection including the pretracheal and paratracheal nodes, precrioid nodes, and the perithyroidal nodes including the lymph nodes along the recurrent laryngeal nerves 9.

### Grouping

There were 777 patients with bilateral lesions and 3330 patients with unilateral lesions. All the bilateral lesions were regarded as independent lesions and a total of 4884 lesions included in the group. The patients who underwent lymph node dissection were grouped according to Gender, Age, Tumor size, Tumor number, Lobe dissemination, Psammoma body, Bilateral, Capsule invasion (Table 1). Tumor size was decided by the maximum diameter of the tumor by the preoperative ultrasonoscopy. Tumor number was decided by the preoperative ultrasonoscopy. Capsule invasion, Lobe dissemination, Psammoma body data for Predictive Risk-scoring Model were based on intraoperative frozen-section examination results and confirmed by paraffin-section post-operation.

**Table 1:** Correlation between Clinical Factors and Central Lymph Node Metastasis(CLNM).

Central Lymph Node Metastasis in CN0 patients					
	−	+	Case number	Positive rate	*P* value
Gender					0.000
Male	463	446	909	49.06%	
Female	2085	1113	3198	34.80%	
Age (years)					0.000
≤25	50	110	160	68.75%	0.000*
25–35	322	339	661	51.29%	
35–45	785	472	1257	37.55%	
45–55	880	425	1305	32.57%	
55–65	415	165	580	28.45%	
>65	96	48	144	33.33%	
Tumor size (cm)					0.000
≤0.5	1570	322	1892	17.02%	0.000**
0.5–1.0	1094	634	1728	36.69%	
1.0–1.5	316	306	622	49.20%	
1.5–2.0	113	168	281	59.79%	
>2.0	132	229	361	63.43%	
Lobe dissemination					0.000
−	3124	1448	4572	31.67%	
+	101	211	312	67.63%	
Psammoma body					0.000
−	3198	1593	4791	33.25%	
+	27	66	93	70.97%	
Tumor number					
solitary	2597	1229	3826	32.12%	0.000
Multifocality	628	430	1058	40.64%	
Bilateral					0.598
−	2207	1123	3330	33.72%	
+	1018	536	1554	34.49%	
Capsule invasion					0.000
−	1988	691	2679	25.79%	
+	1237	968	2205	43.90%	

*Age ≤35 years versus age >35 years.

** Φ ≤ 0.5 cm versus Φ > 0.5 cm.

### Follow-up and postoperative treatment

The time points of follow-up were 3, 6, 9, 12, 18, 24, 36, 48 and 60 months after surgery.

Recurrence was defined as any new lesions detected clinically or radiographically after surgery.

Disease-free survival (DFS) was the duration started from the surgery to recurrence or to the date of most recent follow-up. Overall survival (OS) was the duration started from the surgery to death or to the date of most recent follow-up.

TSH suppression therapy was conventionally performed in PTC patients postoperatively. Postoperative RAI ablation therapy would be performed in patients with total thyroidectomy and one or more of the following conditions: (i) T3 or T4; (ii) positive lateral lymph node metastasis; (iii) distant metastasis. Patients underwent a conventional US examination every 3 months to detect local recurrence and computer tomography (CT) examination of chest every year to detect lung metastasis. Every recurrence in remnant thyroid gland or regional lymph nodes was diagnosed by US and confirmed by fine needle aspiration or histological examination post-operation. Distant metastasis was diagnosed by CT and RAI scintigraphy after completion total thyroidectomy.

### Statistics analysis

Statistical Package for Social Sciemces (SPSS, Inc, Chicago, IL, USA) was used for Statistics analysis. Univariate analysis was performed using chi-square criterion while multivariate analysis was performed using logistic regression analysis. Multiple testing issue was considered when age and tumor size were compared in several groups. A scoring model was constructed to further display the relationships among factors and probability of CLNM. The score of each risk factor was weighted according to the beta coefficient obtained from the logistic regression model. For convenience, all the beta coefficient divided the least one and then rounded to the nearest whole number to keep the scoring model simple. The total score for each patient represented the sum of scores for each risk factor and a risk-scoring model was built to predict the stratified CLNM in PTC patients. To evaluate the predictive performance of the scoring model and find a appropriate cut-off point, we adopted the receiver operating characteristic curve (ROC curve) and evaluated it by the area under the ROC curve (AUROC). Kaplan Meier curves were performed before Cox proportional hazard models. The log-rank test was used to tell the differences of recurrence statistically. A Cox regression model was used to determine prognostic factors. A difference was considered statistically significant when p  < 0.05 and in factors compared.When comparing multiple groups, the new P value was considered to be 0.05/group number.

## Results

### Baseline characteristics

Among all patients, 37.96% (1559/4107) of patients and 33.96% (1659/4884) of lesions confirmed with histologically positive central lymph node metastasis (CLNM). A total of 909 males and 3198 females in the study with the male/female ratio of 1:3.52; the age of the patients ranged from 12 to 82 years with a median age of 45.21 years; the diameter of the tumors ranged from 0.1 cm to 8.0 cm with a median diameter of 0.92 cm. The median follow-up time was 45.00 months (range from 24–143.0 months).

### Risk factors for CLNM

In the univariate analysis, Gender, Age, Tumor size, Lobe dissemination, Psammoma body, Tumor number and Capsule invasion were significantly associated with CLNM (P < 0.05), while no significant correlation was found between Bilateral and CLNM (P > 0.05) (Table 1). In the multivariate analysis, Male (P < 0.01, odds ratio 1.706, 95% CI 1.469–1.981), Age ≤35 years (P < 0.01, odds ratio 2.217, 95% CI 1.893–2.597), Tumor size >0.5 cm (P < 0.01, odds ratio 3.154, 95% CI 2.706–3.676), Lobe dissemination (+) (P < 0.01, odds ratio 3.027, 95% CI 2.333–3.928), Psammoma body (+) (P < 0.01, odds ratio 3.158, 95% CI 1.943–5.132), Multifocality (P < 0.01, odds ratio 1.542, 95% CI 1.321–1.800) and Capsule invasion (+) (P < 0.01, odds ratio 1.508, 95% CI 1.316–1.728) were independent risk predictors of CLNM (Table 2).

**Table 2:** Multivariate logistic regression for central lymph node metastasis.

	*B*^a^	SE^b^	Sig.^c^	Exp(B)^d^	95.0% CI^e^ Exp(B)^d^	
					down	up
Gender(male *vs* female)	0.534	0.076	0.000	1.706	1.469	1.981
Age(≤35 years *vs* > 35 years)	0.796	0.081	0.000	2.217	1.893	2.597
Tumor size (Φ > 0.5 cm *vs* Φ ≤ 0.5 cm)	1.149	0.078	0.000	3.154	2.706	3.676
Lobe dissemination (positive *vs*. negative)	1.108	0.133	0.000	3.027	2.333	3.928
Psammoma bady (positive *vs*. negative)	1.150	0.248	0.000	3.158	1.943	5.132
Capsule invasion (positive *vs*. negative)	0.411	0.069	0.000	1.508	1.316	1.728
Bilateral (positive vs. negative)	0.073	0.072	0.312	1.076	0.933	1.240
Tumor number (Multifocality *vs*. Solitary)	0.433	0.079	0.000	1.542	1.321	1.800
Constant	−1.354	0.096	0.000	0.258		

^a^B: regression coefficient.

^b^SE: Standard Error.

^c^Sig: significance.

^d^Exp(B): odds ratio.

^e^CI: confidence interval.

The rate of CLNM increased obviously with the decrease of age in a certain range and there were significant difference in the rate of CLNM between groups with age ≤25 years, 25 years < age ≤35 years and 35 years < age ≤45 years (P < 0.001). Besides, the rate of CLNM was found to fluctuate strongly between the group with 25 years < age ≤35 years, and 35 years < age ≤45 years (51.29% vs 37.55%) and become relative stable when age ≥35 years, which indicate that maybe age = 35 years could be used as a cut-off point to predictive CLNM. Thus, we regroup the patients and found the CLNM rate was significantly higher in the group age ≤35 years than age >35 years (P < 0.01) (Table 1).

**Figure 1:**
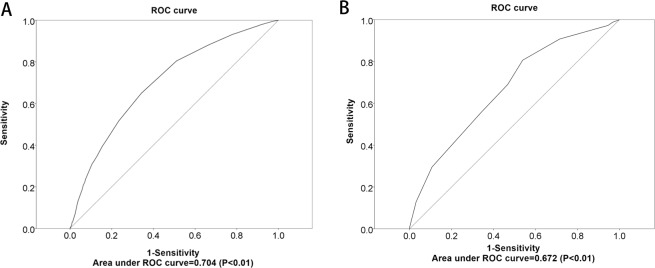
(**A**) ROC curve of the ability of tumor size to predict the likelihood of metastasis to central lymph nodes. (**B**) ROC curve of the ability of risk-scoring model to predict the likelihood of metastasis to central lymph nodes.

Patients were divided into 5 groups based on the maximum diameter of tumor. Significant difference in the rates of CLNM was found between different groups and the rate of CLNM increased obviously with the increase of tumor size. The CLNM rate of the group with Φ < 0.5 cm was significantly lower than other groups. In order to found a cut-off point to predictive CLNM, an ROC analysis was done and the area under the curve was 0.704 (P < 0.01), indicating that Φ = 0.5 cm could be considered a threshold to predict CLNM according to the curve (Fig. 1A).

### Development of risk-scoring model to predict CLNM

A 14-point risk-scoring model was built to predict the stratified CLNM in PTC patients (Table 3). With the scoring model, the rate of positive CLNM ranged from 16.94% to 100% in order of total score in the PTC patients (Table 4). According to the ROC curve, the AUROC of the model for the prediction of CLNM was 0.672 (95% CI: 0.656–0.688) (P < 0.01) (Fig. 1B), which implies the discriminative power of the model is acceptable. Besides, total score = 4 was selected as the appropriate cut-off point of the model and the patients with total score range from 0–3 were divided into low-risk group of CLNM(average CLNM rate: 17.75%), while the patients with total score range from 4–14 were divided into high-risk group of CLN,(average CLNM rate: 43.50 %).

**Table 3:** Development of an 14-point risk-scoring model to predict cervical lymph node metastasis in papillary thyroid carcinoma patients.

	Sig.	Beta coefficient	Point
Gender			
Female			
Male	0.000	0.534	1.000
Age			
>35 years			
≤35 years	0.000	0.796	2.000
Tumor size			
Φ ≤ 0.5 cm			
Φ > 0.5 cm	0.000	1.149	3.000
Lobe dissemination			
negative			
positive	0.000	1.108	3.000
Psammoma bady			
negative			
positive	0.000	1.150	3.000
Capsule invasion			
negative			
positive	0.001	0.411	1.000
Tumor number			
solitary			
multifocality	0.000	0.433	1.000

**Table 4:** Risk scores and percentage of positive central lymph node metastasis in papillary thyroid carcinoma patients.

Risk score	Negative	Positive	Total	Positive rate
0	104	20	124	16.13%
1	70	25	95	26.32%
2	744	108	852	12.68%
3	569	168	737	22.80%
4	230	193	423	45.63%
5	404	219	623	35.15%
6	760	436	1196	36.45%
7	244	276	520	53.08%
8	41	84	125	67.20%
9	32	64	96	66.67%
10	24	49	73	67.12%
11	3	11	14	78.57%
12	0	4	4	100.00%
13	0	2	2	100.00%

### Predictors of recurrence in patients with PTC

A total of 4107 patients were followed up and the average follow-up time was 49.90 months (standard deviation: + /−23.91 months) and the median follow-up time was 45 months (range from 24–143 months).One hundred patients (2.43%) were found with recurrence and 25 (0.61%) patients were dead, in which only 9 (0.22%) were attributed to PTC. Thus, we evaluated the disease-free survival instead of overall survival due to the small number of patients with death. COX regression model showed that Tumor size >0.5 cm (P < 0.01,hazard rate 2.601, 95% CI 1.382–4.896), Lobe dissemination (+) (P < 0.01,hazard rate 2.649, 95% CI 1.630–4.305), Multifocality (P < 0.01,hazard rate 3.070, 95% CI 1.973–4.788) and CLNM (P < 0.01,hazard rate 2.914, 95% CI 1.728–4.912) were risk predictors of recurrence. (Table 5).

**Table 5:** Log-rank test and Cox’s proportional hazards regression model for recurrence.

Variable	Univariate analysis		Multivariate analysis		
	Sig.^a^	DFS rate(%)	Sig.^a^	Exp(B)^b^	95.0% CI^c^
Gender (male vs female)	0.054	96.7% vs 97.8%	0.183	1.340	0.871–2.061
Age (≤35 years vs > 35 years)	0.658	97.7% vs 97.4%	0.444	1.171	0.782–1.754
Tumor size (Φ > 0.5 cm vs Φ ≤ 0.5 cm)	0.000	96.7% vs 99.2%	0.003	2.601	1.382–4.896
Lobe dissemination (positive vs. negative)	0.000	91.2% vs 98.0%	0.000	2.649	1.630–4.305
Psammoma bady (positive vs. negative)	0.006	93.1% vs 97.7%	0.121	1.943	0.839–4.497
Capsule invasion (positive vs. negative)	0.019	97.1% vs 98.0%	0.548	0.879	0.578–1.338
Bilateral (positive vs. negative)	0.002	96.2% vs 97.9%	0.128	0.677	0.410–1.118
Tumor number (Multifocality vs. Solitary)	0.000	94.9% vs 95.8%	0.000	3.070	1.973–4.778
CLNM (positive vs. negative)	0.000	95.5% vs 99.1%	0.000	2.914	1.728–4.912

^a^Sig: significance.

^b^Exp(B): odds ratio.

^c^CI: confidence interval.

**Figure 2:**
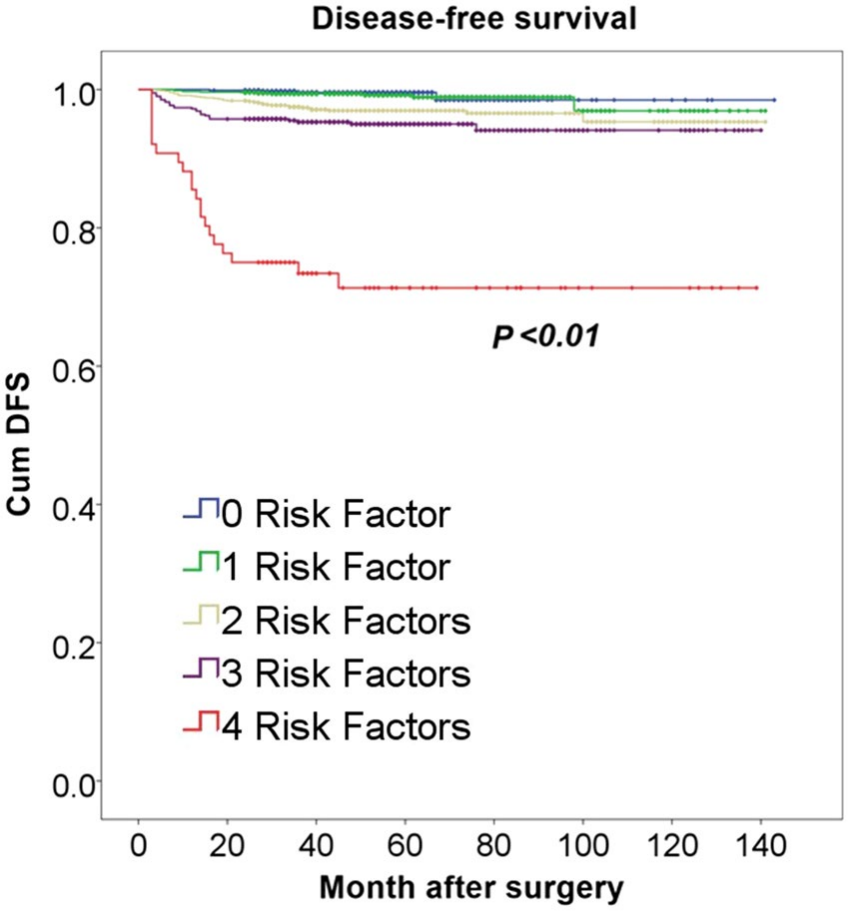
DFS rate in 4-factors group was significantly lower than other groups (P < 0.01).

As was shown above, Tumor size >0.5 cm, Lobe dissemination (+), Multifocality and CLNM were significant risk factors associated with poor outcomes. Thus, we regrouped the patients into five groups according to the number of these risk factors. There were 889 patients with 0-factor, 1364 patients with 1-factor, 1168 patients with 2-factors, 610 patients with 3-factors and 76 patients with 4-factors. There were significant differences in disease-free survival (DFS) rate between the five groups. The DFS rate was highest in 0-factor (99.60% at 143 months) and 1-factors (99.20% at 143 months), lowest in 4-factors group (72.40% at 143 months), and intermediate in 2-factors group (97.10% at 143 months) and 3-factors group (95.10% at 143 months) (Fig. 2). Patients with a greater number of risk factors were usually more vulnerable to recurrence.

## Discussion

The aim of this study was to evaluate the predictive factors of CLNM and identify risk factors of recurrence in the Han Chinese. There were about 37.96% of PTC patients were proved with CLNM by histopathologic result post-operation, which means that more than one third of the patients would suffer from incomplete treatment if we did not perform prophylactic CLN dissection in PTC patients. The occult metastatic lymph nodes would be remained in the bodies and became one of the most important sources of recurrence. Our data showed that the recurrence rate of PTC was 2.43% (100 patients) and only 9 were died of PTC during follow-up, which implied a good prognosis of PTC patients. The main reason of recurrence may be due to incomplete CLN dissection or occult lateral cervical lymph node metastasis. Thus, we considered that even in the patients underwent prophylactic CLN dissection, there were still 2.43% of patients suffer from recurrence. Then, there will be a higher rate of recurrence in patients without prophylactic CLN dissection. Then how would be the patients without prophylactic CLN dissection?

Currently, an increasing number of experts supported prophylactic central neck dissection in PTC patients for the reasons as follows^11^: (i) Lymph node metastases have a negative effect on patients’ prognosis. (ii) prophylactic CLN dissection contributes to remove subclinical metastasis and reduce recurrence. (iii) prophylactic CLN dissection does good to the follow-up treatment, including more accurate staging, prognosis evaluation and radioactive iodine therapy. (iv) central neck dissection can be achieved reliably. (v) for central neck recurrence, reoperation leads to elevated morbidity. Prophylactic CLN dissection was also recommended by the American Thyroid Association (ATA) Guidelines for PTC, particularly for patients who have advanced primary tumors (T3 orT4)^7^. However, Kim SK *et al*.^12^ suggested that prophylactic central neck dissection may not be proposed in PTC due to the absence of proven benefit and clear evidence of morbidity, for instance temporary vocal cord palsy and temporary hypoparathyroidism. We support the former for the high rate of occult CLNM and its negative effect on recurrence. Besides, as a retrospective study, there was an inevitable serious selection bias in Kim's work, which may affect the credibility of the results.

It has been wildly accepted that women were more susceptible to PTC. Grebe SK and Hay ID revealed the notable relevance between the morbidity of PTC and estrogen level in women^6^ and Fan D *et al*.^13^ considered that estrogen receptor α could promote PTC by improving autophagy in PTC cells, a vital pro-survival catabolic way. An entirety of 909 males and 3198 females in our research in which the male: female ratio(1:3.52) is consistently comparing with our former studies. Moreover, our results showed that the CLNM rate of male (49.06%) was significantly higher than female (34.80%) (P < 0.01, odds ratio 1.706), which is inconsistent with our previous study^14^. The main reason of the discrepancy may be the more extensive sample size of this study. However, in Cox regression analysis, male is not the risk factor of recurrence.

Age has been used as a prognosis predictor in PTC patients for many years and it is one of the most important factors to be considered to identify the PTC patients with high risk or low risk based on the TNM classification^15^. But its role in CLNM was still controversial. Some scholars found no significant diversity in the older cases (age >45 years) comparing to older cases (age >45 years) on CLNM^16^. In contrast, Siddiqui S *et al*.^17^ argued that CLNM rate in young group (age ≤45 years) was notably higher contrasted to older ones (age >45 years) in Papillary Thyroid Microcarcinoma (63.4% vs. 36.6%, P = 0.029). Our results supported the latter. 6 groups were divided on the basis of age (per 10 years) for further study and it has been found that the rate of CLNM was apparently inferior to the group whose age ≤35 years than those whose age >35 years (54.69% vs.33.78%, P < 0.01, odds ratio 2.217) and became relative stable when age ≥35 years, which indicated that patients with less than 35 years old were more vulnerable to CLNM and age = 35 years maybe could be used as a cut-off point to predict CLNM. Otherwise, no significant correlation was found between age and recurrence.

Some studies reported that patients with tumor size >3 cm would be more susceptible to recurrence in the lymph node^18^. Qn U *et al*.^19^ discovered tumor size was highly associated with the number of CLNM. We found that increasing CLNM was related to the enlarging tumor size (P < 0.01) and Φ = 0.5 cm was presented as a cut-off point for prediction of CLNM by ROC curve (Fig. 1). This result was incompatible with a previous study suggesting tumor size was independent of CLNM using logistic regression^17^, which may be due to the more extensive sample size in this study than previous. Furthermore, it was found in our study that tumor size exceeding 0.5 cm presented a higher risk of recurrence (P < 0.01).

Capsule invasion was always considered as a symbol of tumor progression and patients with capsule invasion appeared to be more prone to attack and recurrence^20^. Extension involving extrathyroidal was found associate with lower surviving rate^21^ and maybe one of the risk factors increasing lymph node metastasis^22^. Extrathyroidal extension was also considered to have a positive effect on the number of CLNM^23^. Our results showed that capsule invasion is one of the independent predictors for CLNM (P < 0.01), while no meaningful correspondence was observed between capsule invasion and recurrence, which indicated that microscopic extent invasion of thyroid capsule may not affect the recurrence of the patients.

Recently, Kim HJ^24^ and colleges suggested that an increasing amount of tumor foci was significantly correlated with cervical lymph node metastasis and advanced TNM stage of PTC and the number of tumor foci independently predicted lateral lymph node metastasis. Increased tumor number seemed to be more likely to happen capsular invasion, extrathyroidal extension, and lymph node metastasis^25^. Previous studies showed that psammoma body is beneficial to predict prognosis and adverse clinical biological behavior of PTC^26,27^. Can N^28^ *et al*. considered that intraglandular dissemination, lateral tubular growth, tumor border and lymphocytic/stromal tumor response, multifocality, concomitant lymphocytic thyroiditis were easier to have lymph node metastasis. In this study, intraglandular dissemination (+), psammoma body (+) and multifocality were independent risk factors of CLNM and intraglandular dissemination (+) and multifocality may shorten the prognosis of PTC patients.

Subsquently, based on the independent risk factors mentioned above, we developed a risk-scoring model to predict CLNM. It is well known that a predictive model not only enlightens each predictor on the probability of a response from levels of other factors but also provides a quick estimation of the probability from response for individual subjects. This risk-scoring model shows good discriminative power. The average CLNM rate of the patients with score ≥4 was significantly higher than the patients with score ≤3 (43.50% vs. 17.75%, P < 0.01). The risk-scoring model was very simple and efficient to implement in clinical works and could preliminarily help surgeons to identify high-risk groups in a relatively objective way. With this model, combining with image examinations, surgeons could make a more comprehensive and accurate decision pre/intra-operation to avoid the incomplete treatment in the central compartment. Due to the high-risk of CLNM, we advocated prophylactic CLN dissection in patients with total score ≥4 according to the risk-scoring model.

The results of our study showed that Tumor size >0.5 cm, Lobe dissemination (+), Multifocality and CLNM were significant risk factors for recurrence. The DFS rate of patients having 4-factors was notably more moderate than other groups, indicating that patients with a great deal of risk factors were generally more vulnerable to recurrence and more complete treatment as well as a more frequent follow-up should consider for patients with 4 factors.

However, the present study has some certain limitations, which expects to be extended upon in additional researches. Firstly, as a retrospective study, selection bias is inevitable in this study. Secondly, part of patients had unilateral lesions, who underwent unilateral lobectomy plus isthmusectomy and ipsilateral CLN dissection in this study, while we don’t know whether those patients have occult contralateral thyroid tumors. Thirdly, all the patients included were Chinese. It is not certain that the conclusion applies to other races.

## Conclusion

In summary, more than one third of PTC patients were proved with CLNM in our research, which indicated the generalization of CLNM in PTC. Patients with male, age ≤35 years, tumor size >0.5 cm, Lobe dissemination (+), Psammoma body (+), Multifocality or Capsule invasion (+) were considered more vulnerable to CLNM, and CLNM, Tumor size >0.5 cm, Lobe dissemination (+) and Multifocality may increase the risk of recurrence and perhaps decrease survival. On this account, according to our model, we suggested that prophylactic CLN dissection could be performed routinely in PTC patients with total score ≥4, and more aggressive treatment and more frequent follow-up should be considered for patients with CLNM, Tumor size >0.5 cm, Lobe dissemination (+) and Multifocality.

## Data Availability

The datasets used and analyzed during the current study are available from the corresponding author on reasonable request.
